# Network Analysis of the Brief ICF Core Set for Schizophrenia

**DOI:** 10.3389/fpsyt.2022.852132

**Published:** 2022-06-17

**Authors:** Laura Nuño, Georgina Guilera, Maite Barrios, Juana Gómez-Benito, Gomaa Said Mohamed Abdelhamid

**Affiliations:** ^1^Addictions Unit, Clinical Institute of Neuroscience (ICN), Hospital Clinic, Barcelona, Spain; ^2^Department of Social Psychology and Quantitative Psychology, University of Barcelona, Barcelona, Spain; ^3^Group on Measurement Invariance and Analysis of Change (GEIMAC), Institute of Neurosciences, University of Barcelona, Barcelona, Spain; ^4^Department of Educational Psychology, Faculty of Education, Fayoum University, Fayoum, Egypt

**Keywords:** network analysis, ICF core set, schizophrenia, Delphi method, functioning

## Abstract

**Background:**

The International Classification of Functioning, Disability, and Health Core Sets (ICF-CSs) for schizophrenia are a set of categories for assessing functioning in persons with this health condition. This study aimed to: a) estimate the network structure of the Brief ICF-CS for schizophrenia, b) examine the community structure (categories strongly clustered together) underlying this network, and c) identify the most central categories within this network.

**Methods:**

A total of 638 health professionals from different backgrounds and with a significant role in the treatment of individuals with schizophrenia participated in a series of Delphi studies. Based on their responses we used the Ising model to estimate the network structure of the 25-category Brief ICF-CS, and then estimated the degree of centrality for all categories. Finally, the community structure was detected using the walktrap algorithm.

**Results:**

The resulting network revealed strong associations between individual categories within components of the ICF (i.e., *Body functions, Activities and participation*, and *Environmental factors*). The results also showed three distinct clusters of categories corresponding to the same three components. The categories *e410 Individual attitudes of immediate family members, e450 Individual attitudes of health professionals, d910 Community life*, and *d175 Solving problems* were among the most central categories in the Brief ICF-CS network.

**Conclusion:**

These results demonstrate the utility of a network approach for estimating the structure of the ICF-CSs. Implications of these results for clinical interventions and development of new instruments are discussed.

## Introduction

Schizophrenia is a chronic and disabling mental disorder that is characterized by heterogeneous symptoms, both positive (e.g., delusions and disorganized thinking) and negative (e.g., blunted affect and anhedonia), as well as cognitive impairment and multiple functional deficits (1, 2). Although recovery is possible and should be a priority goal in the treatment of this population, it remains a challenge insofar as persons diagnosed with schizophrenia usually experience important disability in personal, social, and occupational functioning across their life span (3, 4). Nevertheless, research suggests that interdisciplinary mental health teams providing integrative care to individuals with schizophrenia can achieve substantial improvements in clinical, social, and health outcomes (5, 6). However, providing integrated care requires a common language that enables interdisciplinary team members to develop a shared understanding of a patient's functioning problems. The International Classification of Functioning, Disability, and Health (ICF), which was proposed and adopted in 2001 by the World Health Organization (WHO) (7), meets these requirements.

The WHO conceptualizes health in terms of a biopsychosocial model and uses the term *functioning* to refer to the positive and practical aspects of health, that is to say, what a person can or cannot do in daily life, regardless of any diagnosed disease or specific health condition (7). It was within this conceptual framework that the ICF was developed. The main objectives of the ICF are to provide a scientific basis for the understanding and description of health and health-related states, to establish a unified and standard language to describe them, and to permit comparison of data across countries, health care disciplines, services, and over time (7). The ICF describes functioning in persons with any health condition through the dynamic interaction between the following components: *Body functions*, which comprise the physiological and psychological functions of the body systems; *Body structures*, which refer to the anatomical parts of the body; *Activities and participation*, encompassing the execution of all the tasks and actions that an individual may perform and which may be involved in a life situation; and *Contextual factors*, which includes both *Environmental* and *Personal factors*. Regarding the latter component, *Environmental factors* reflect the physical, social, and attitudinal environment in which individuals live and conduct their lives, whereas *Personal factors* refer to the particular background of an individual's life and comprise traits that are independent of his or her health condition, such as gender, race, or age.

The ICF system comprises more than 1,400 categories. To facilitate its implementation, ICF Core Sets (ICF-CSs) linked to specific health conditions have been developed using a protocol proposed by the WHO (8); those developed to date can be viewed and downloaded at: https://www.icf-core-sets.org/en/page1.php. The ICF-CSs consist of a list of the most relevant ICF categories for describing and assessing functioning and disability in people living with a certain health condition, and in most cases both a comprehensive and a brief version of the Core Set have been developed. The categories listed in a Comprehensive ICF-CS cover the full spectrum of problems in functioning that are typically experienced by individuals with a specific health condition; the corresponding Brief ICF-CS consists of a selection of the most essential categories that should be considered when exploring functioning in individuals with this health condition (8). Thus, ICF-CSs could serve as a reference pool of categories to identify individual's functional strengths and weaknesses, plan appropriate interventions, and develop standardized assessment instruments. For schizophrenia, two ICF-CSs (i.e., the Comprehensive and the Brief version) have been developed following the evidence-based process proposed by the ICF Research Branch, a WHO collaborating center (9). The Comprehensive ICF-CS for schizophrenia comprises 97 categories representing a broad spectrum of common problems in functioning suffered by persons with schizophrenia. The corresponding Brief ICF-CS includes 25 of these 97 categories, those of most importance in the assessment and treatment of persons with schizophrenia and hence of most relevance to clinical practice. The two ICF-CSs for schizophrenia can be viewed and downloaded free of charge at: https://www.icf-research-branch.org/icf-core-sets-projects2/mental-health/icf-core-set-for-schizophrenia.

In order to be applicable in clinical practice, ICF-CSs must be validated through different sources of evidence. Evidence for the content validity of the ICF-CSs for schizophrenia has been obtained in a series of previous studies in which we used the Delphi method to explore the perspective of different health professionals involved in treating persons with schizophrenia, namely psychiatrists (10), psychologists (11), nurses (12), occupational therapists (13), social workers (14), and physiotherapists (15). The results of these studies indicated that both the Comprehensive and Brief ICF-CSs for schizophrenia provide an effective framework for investigating functioning and disability in persons with schizophrenia. However, the ways in which the different components of functioning in schizophrenia may interact with one another has yet to be tested empirically. To address this gap, the present study uses network analysis to analyze the data obtained in the aforementioned series of Delphi studies.

To the best of our knowledge, no study has applied network analysis to examine the structure of ICF-CSs, including the Brief ICF-CS for schizophrenia. The aim of the present study was therefore to: (1) estimate the network structure of the Brief ICF-CS for schizophrenia and examine the connections between its categories using data obtained from different health professionals with experience in the assessment and/or treatment of individuals with schizophrenia; (2) examine the community structure (categories strongly clustered together) underlying this network; (3) identify the most central ICF categories that are associated with functioning and disability within this network; (4) identify the bridge categories (i.e., the categories in a component that have strong edges with all categories in all other communities, and vice versa); and (5) assess the robustness and stability of this network.

## Methods

### Data Collection

In a previous series of three-round Delphi studies we explored the perspective of professionals from six different health disciplines (i.e., psychiatry, psychology, nursing, occupational therapy, social work, and physiotherapy) regarding the individual problems, resources, and environmental factors (presented in the form of ICF categories) that they most frequently encounter when treating individuals with schizophrenia. The Delphi method is a multistage process in which a panel of experts are asked to give their opinion about a specific topic across a series of rounds. After each round, each panel member receives feedback in the form of an anonymous summary of the responses given by the other experts, which they must take into account before giving their opinion again (16). This methodology allows researchers to obtain the opinion of numerous experts on the same subject with the objective of reaching a consensus (17).

**Figure 1:**
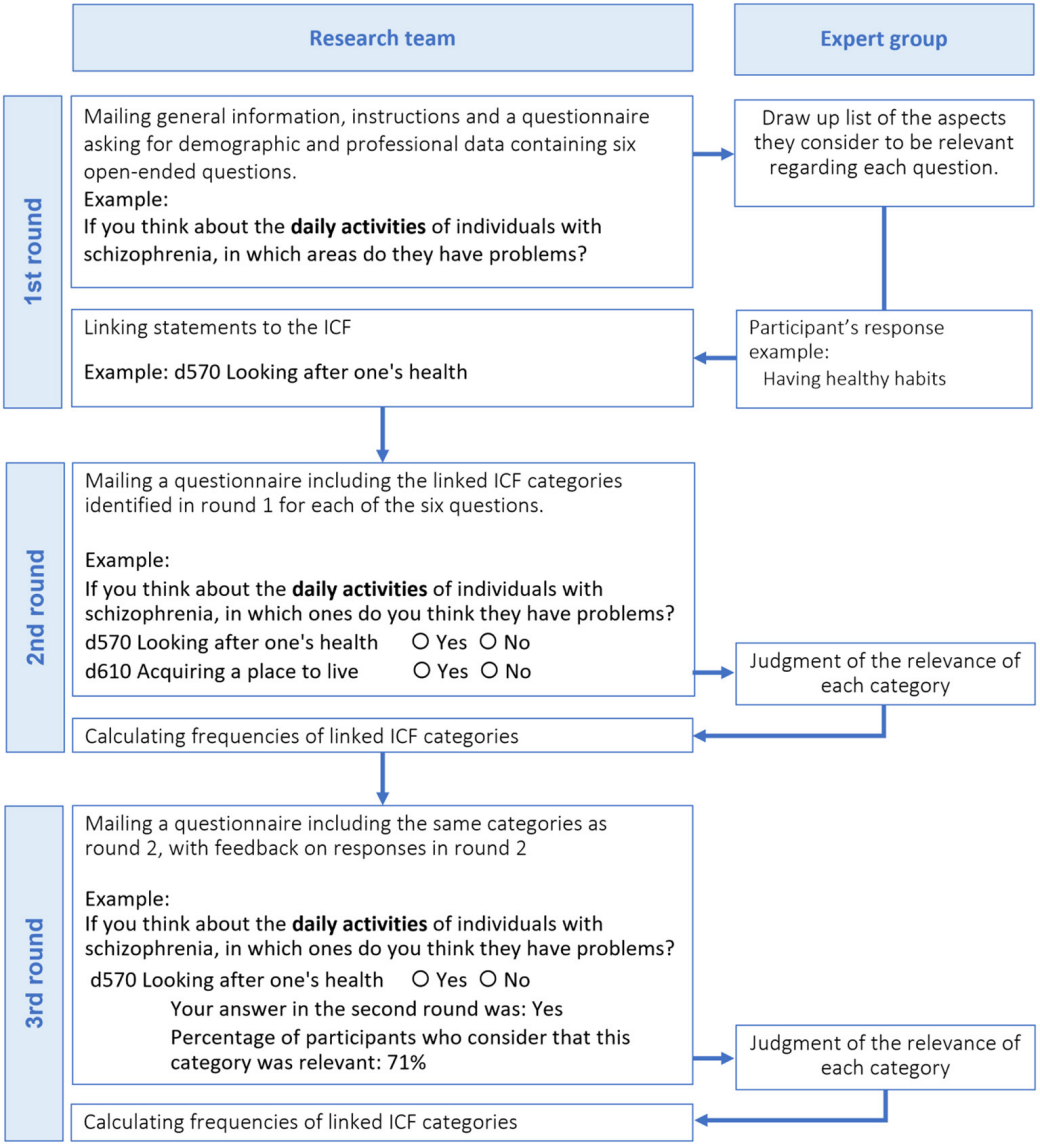
Process followed in the Delphi studies.

A total of 790 health professionals from 85 different countries representing all six WHO regions (i.e., Africa, The Americas, Eastern Mediterranean, Europe, South-East Asia, and Western Pacific) participated in the first round of the six aforementioned Delphi studies, and of these, 638 completed all rounds of the Delphi process. The recruitment of participants and the Delphi process are described in detail in Nuño et al. (18) and summarized in Figure 1. The task for participants in these studies was to judge, from their professional perspective, whether they considered each ICF category to be relevant or not (yes/no) to the assessment and/or treatment of persons with schizophrenia.

### Network Analysis

The data obtained from the six Delphi studies were used to estimate the network structure of the Brief ICF-CS for schizophrenia. From the network perspective, the ICF categories rated by experts may be represented as nodes (circles), which are connected with edges (lines) when they tend to co-occur (i.e., they are selected as relevant by the same expert), forming a network structure. Applying this approach, the 25 categories of the Brief ICF-CS for schizophrenia produced a network with 25 nodes (i.e., categories) and 300 potential edges (i.e., connections) between these nodes. Network analysis was conducted following the methodology described by Epskamp et al. (19).

### Network Estimation

Given the binary nature of the variables, the Ising model (20) was used to estimate the Brief ICF-CS network. In this model, the edges (i.e., connections) between nodes (i.e., ICF categories) are estimated using regularized logistic regression (20). These edges can be understood as partial correlation coefficients, which means that a correlation between two nodes A and B is estimated as a conditional dependence relationship after controlling for all other connections between nodes in the network. In other words, if nodes A and B are not connected after controlling for all the connections between all other nodes, then A and B may be considered independent nodes. The Ising model also employs a regularization strategy whereby very small correlations (connections) are shrunk to be exactly zero; this decreases the number of false positive connections between nodes and allows more accurate detection of the underlying network structure (20). The Brief ICF-CS network was estimated using the R *IsingFit* package (version 0.3.1) with gamma = 0.25 (20), and the results were visualized with the *qgraph* package (version 1.6.5) (21). Extensive details regarding the Ising model can be found in van Borkulo et al. (20).

Finally, the community (or cluster) structure of the network was detected using the walktrap algorithm (22). In this case, a community describes a set of categories that are strongly associated/clustered together within the estimated network.

### Node Centrality Indices

The centrality of each category in the network was computed to identify those categories which form the core or are more important than others (23). To this end, we obtained the following three indices: a) the *node strength* represents the sum of the absolute values of all connections with respect to other nodes; b) the *closeness centrality* measures how strongly a node is associated indirectly with other nodes in the network; and c) the *betweenness centrality* assesses the shortest path length connecting any two nodes (19). These three centrality indices were extracted and graphs were generated to investigate the centrality of each of the categories.

### Bridge Centrality

We also estimated the bridge expected influence (BEI; one step) (24) via the *networktools* package (25). The BEI determines potential bridge nodes by summing all absolute edges between a node (e.g., category *b160 Thought functions* from the *Body functions* component of the Brief ICF-CS) and all nodes that do not form part of the same component (i.e., categories from the *Activities and participation* and *Environmental factors* components). Nodes with high absolute values of BEI are potentially important as bridge nodes. For instance, the BEI of a *Body function* category (symptom) indicates to what extent this category is related to categories of the *Activities and participation* and *Environmental factors* components. Identifying these bridge nodes may yield hypotheses about categories/symptoms that cause (or prevent) the occurrence of positive (or negative) outcomes (26).

The parameter accuracy of edges and the stability of centrality indices in the estimated network were examined using the *bootnet* package (version 1.4.3) in R (19), specifically through a bootstrap sampling technique with 1,000 iterations. The accuracy of edges was investigated using the non-parametric bootstrap technique to draw the 95% confidence intervals (CIs) for the edge-weights. Additionally, we used the *case-dropping* bootstrap technique to investigate the stability of the order of nodes in terms of centrality. This technique yields a correlation stability (CS) coefficient, which shows how many cases (i.e., proportion of individuals) might be removed from the analysis while maintaining a correlation of at least 0.7 with the original centrality values within a 95% confidence interval. Consequently, the CS coefficient assesses whether original estimates correlate with bootstrapped estimates. The CS value should not be below 0.25, and ideally it will be above 0.50. The case-dropping bootstrap technique was also used to assess the stability of the bridge. Further information about these methods can be found in Epskamp et al. (19).

## Results

### Sample Description

Of the 638 health professionals who completed the Delphi studies, 303 (47.5%) were psychiatrists, 137 (21.5%) were psychologists, 79 (12.4%) were nurses, 73 (11.4%) were occupational therapists, 36 (5.6%) were social workers, and 10 (1.6%) were physiotherapists. Overall, 52.0% of respondents were male. Demographic and professional characteristics of participants are summarized in Table 1. More detailed information regarding the six expert panels is available in Nuño et al. (18).

**Table 1:** Demographic and professional data for participants who completed the six Delphi studies.

**WHO region**	**Frequency *n* (%)**	**Female** ***n* (%)**	**Experience in schizophrenia mean years (range)**
Africa*^*a*^*	39 (6.1%)	17 (43.6)	12.3 (2–34)
The Americas*^*b*^*	143 (22.4%)	76 (53.1)	18.2 (1–55)
Eastern Mediterranean*^*c*^*	39 (6.1%)	18 (46.2)	10.4 (1–36)
Europe*^*d*^*	206 (32.3%)	133 (64.6)	14.9 (1–45)
South-East Asia*^*e*^*	103 (16.2%)	33 (32.0)	14.2 (1–51)
Western Pacific*^*f*^*	108 (16.9%)	29 (26.9)	18.3 (1–48)
Total	638	306 (48.0)	15.7 (1–55)

a *Ethiopia, Nigeria, South Africa, and Uganda*.

b *Argentina, Brazil, Canada, Chile, Cuba, Mexico, USA, and Venezuela*.

c *Egypt, Iran, Kuwait, Morocco, Pakistan, and Saudi Arabia*.

d *Armenia, Belgium, Bosnia and Herzegovina, Bulgaria, Croatia, Czech Republic, Denmark, Finland, France, Georgia, Germany, Greece, Hungary, Israel, Italy, Latvia, Lithuania, Macedonia, Norway, Poland, Romania, Russia, Serbia, Slovakia, Slovenia, Spain, Sweden, Switzerland, Turkey, and UK*.

e *Bangladesh, India, Indonesia, Nepal, Sri Lanka, and Thailand*.

f *Australia, Cambodia, China, Japan, South Korea, Malaysia, New Zealand, Philippines, and Taiwan*.

### The Brief ICF-CS Network

**Figure 2:**
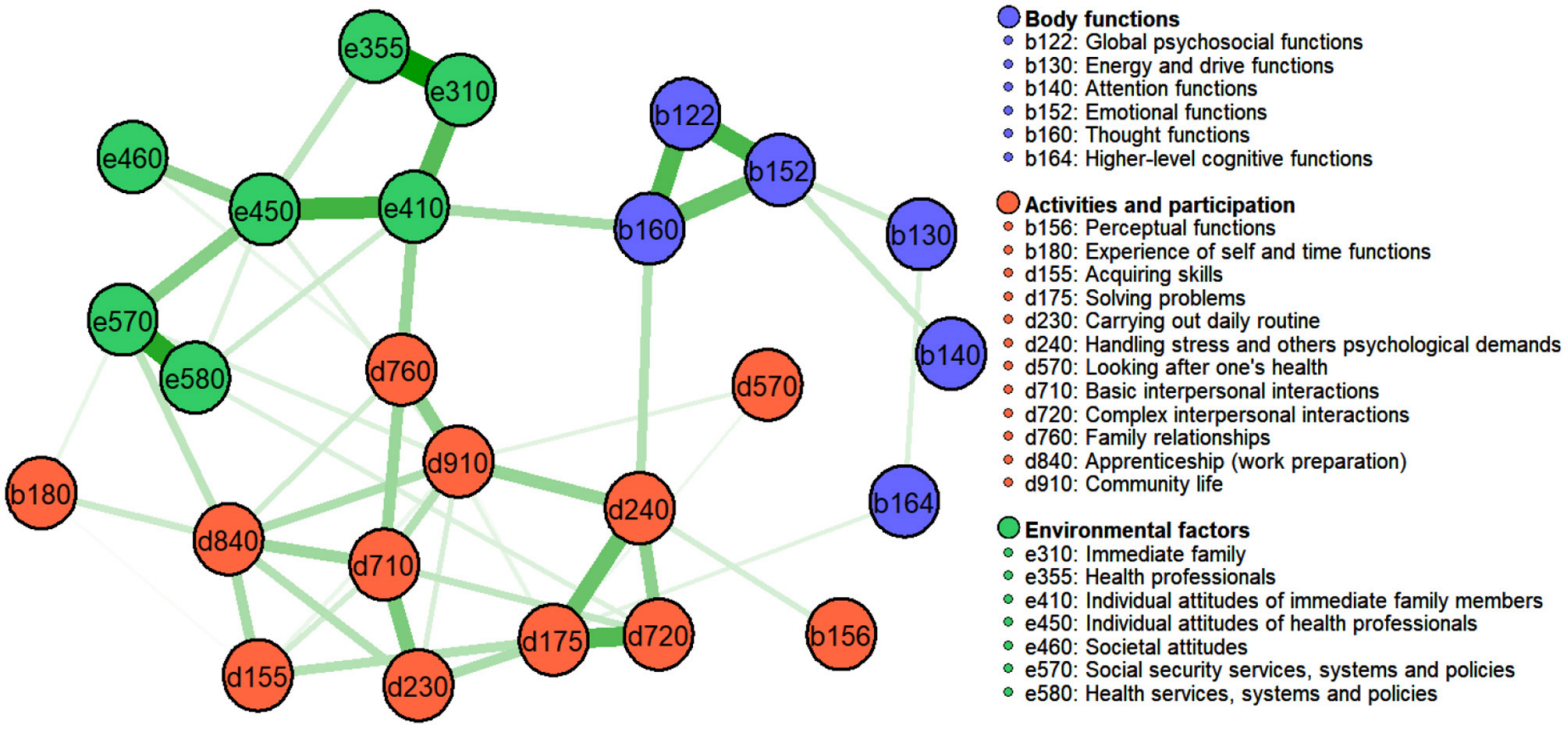
Estimated network for the 25 categories of the Brief ICF-CS for schizophrenia, with the identified communities (walktrap algorithm). Nodes represent ICF categories and edges represent pairwise dependencies between the categories, after controlling for all other correlations of a given node.

Figure 2 depicts the network structure of the Brief ICF-CS for schizophrenia, showing the connections between the 25 ICF categories. Each node in this network represents a category, and each edge represents bidirectional partial relations between categories after controlling for all other associations in the estimated network. There were no unconnected nodes, and 52 of all potential 300 edges were estimated to be above zero, indicating a medium-density network (i.e., 17% of the possible connections were observed in the network). Moreover, all of these connections were positive (solid green edges indicate positive associations, whereas red edges indicate negative associations).

Figure 2 also shows the results obtained when using the walktrap algorithm to detect communities within the Brief ICF-CS network. Closer inspection of this figure indicates three clinically meaningful clusters corresponding to the following ICF components: *Environmental factors* (shown in green: 7 *e* categories), *Body functions* (shown in purple: 6 *b* categories), and *Activities and participation* (shown in red: 10 *d* and 2 *b* categories).

#### Node Centrality Indices

**Figure 3:**
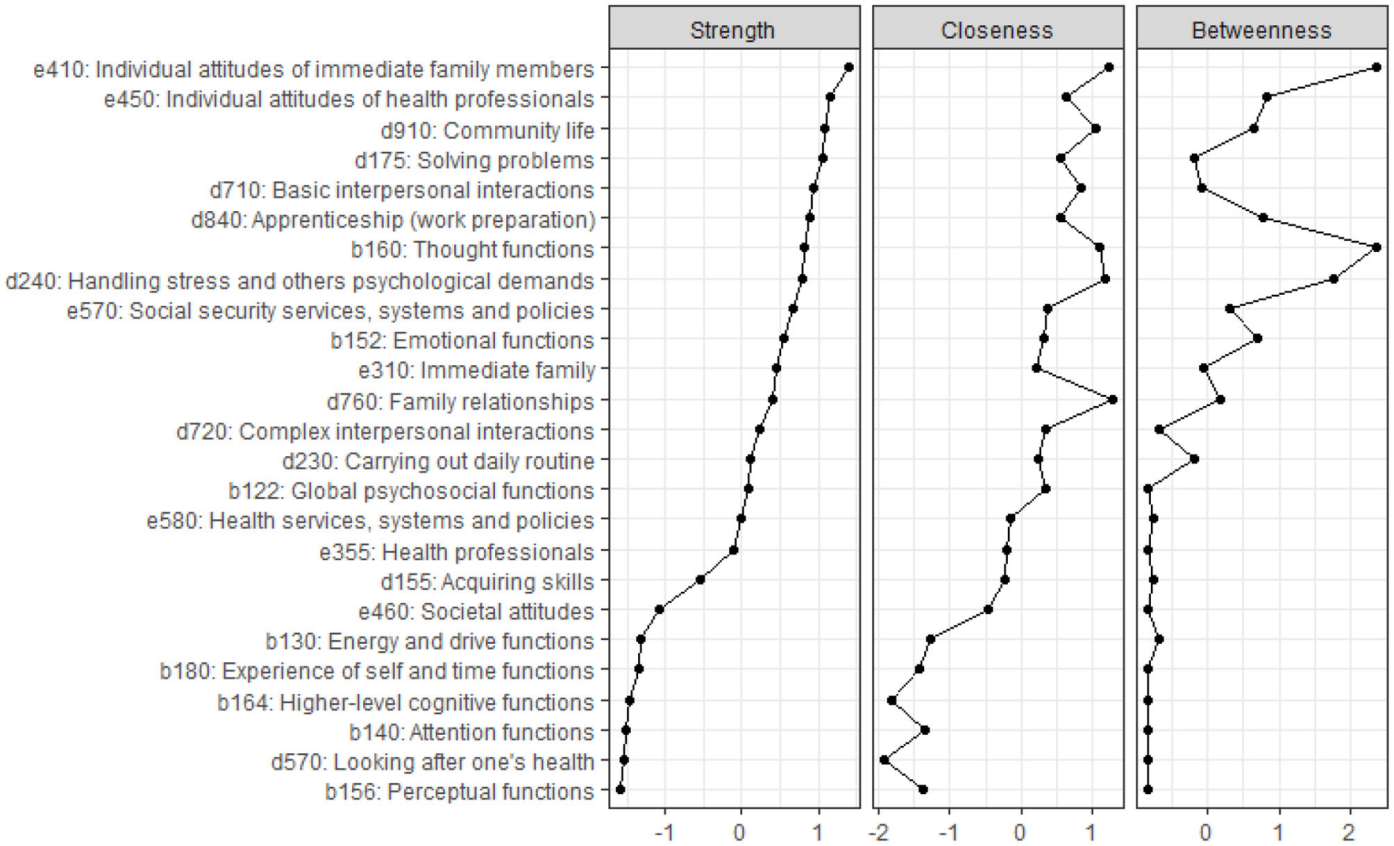
Standardized node strength, closeness, and betweenness centrality indices for each of the 25 categories in the Brief ICF-CS for schizophrenia, ordered by node strength.

Figure 3 presents the standardized estimates of the three centrality measures for each category. These three centrality estimates appear to be highly intercorrelated, with significant correlations being observed between node strength and closeness (0.931), node strength and betweenness (0.724), and betweenness and closeness (0.713). We will therefore focus on node strength as the measure for identifying the most central categories in the estimated network.

Based on node strength, the five most central categories were *e410 Individual attitudes of immediate family members, e450 Individual attitudes of health professionals, d910 Community life, d175 Solving problems*, and d710 *Basic interpersonal interactions*; the least central categories were *b156 Perceptual functions* and *d570 Looking after one's health*. From the network perspective, this implies that these five categories provide (from the point of view of expert professionals) the most important information about problems in functioning among individuals with schizophrenia.

Concerning the accuracy analyses, bootstrapped results were relatively narrow for the estimated edge-weights (i.e., connection weights between the 25 categories; see Supplementary Figure S1), suggesting that the estimated edges were relatively reliable. Regarding the stability of centrality indices, the results from the case-dropping subset bootstrap indicated that the order of node strength centrality was more stable than the order of betweenness and closeness indices when dropping large proportions of the sample (Supplementary Figure S2), although CS coefficients were low (< 0.25) for all indices.

#### Bridge Centrality

**Figure 4:**
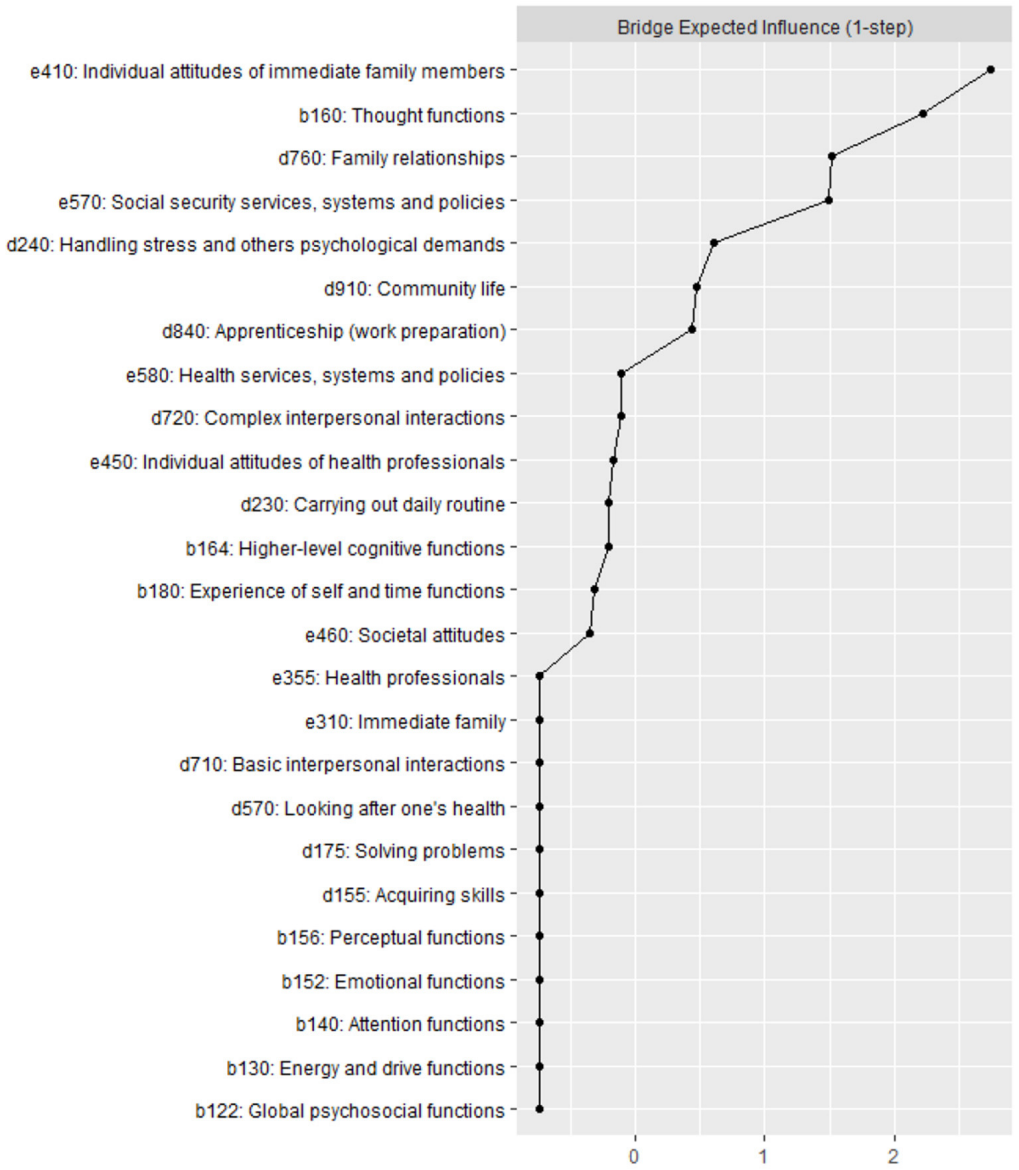
Standardized bridge expected influence (one-step) for each of the 25 categories in the Brief ICF-CS network (as shown in Figure 2).

Figure 4 shows that the standardized BEI is strongest for the categories *e410 Individual attitudes of immediate family members, b160 Thought functions, d760 Family relationships*, and *e570 Social security services, systems and policies* (all with *z* score > 1). However, the findings for bridge stability when using the case-dropping bootstrap method indicated low stability when dropping large proportions of the sample (CS coefficient < 0.25) (see Supplementary Figure S2).

## Discussion

Although many studies have described the problems that people with schizophrenia most frequently experience in daily life (27–30), the present report is the first to apply network analysis to determine the relevance of and interrelationships between these problems from the perspective of health professionals, using the ICF as a conceptual framework. To the best of our knowledge, it is the first time that network analysis has been applied to the Brief ICF-CS for schizophrenia.

Consistent with the ICF model, the network approach depicts functioning as a dynamic system of node-to-node interactions (31), with each node representing an ICF category or aspect of functioning. Accordingly, we sought here to provide a new empirical perspective on the adequacy of ICF categories for describing the functioning of persons with schizophrenia, in this case from the perspective of health professionals from different disciplines (and all six world regions defined by the WHO) with experience in the treatment and/or assessment of people with schizophrenia. In our view, the present study makes four novel contributions to the ICF-CS literature, insofar as we 1) estimate the network structure of the 25-category Brief ICF-CS for schizophrenia, 2) assess the degree of centrality of each of the 25 categories in this network, 3) identify the community structure underlying the Brief ICF-CS network, and 4) investigate the stability and robustness of this network.

Regarding network estimation, our findings largely support the component structure of the Brief ICF-CS as defined by the biopsychosocial model, and as such they broadly corroborate the international validity of the ICF-CSs for schizophrenia from the perspective of these health professionals. Importantly, however, the analysis also identified specific aspects of functioning with high centrality and associations between a wide range of ICF categories within the Brief ICF-CS network, thus indicating the potential importance of these categories in the treatment of individuals with schizophrenia. Although these findings underline the importance of highly specific aspects of functioning that have already been the focus of different programs aimed at improving the functioning of individuals with schizophrenia (32–34), they also provide a novel framework for the design of more comprehensive interventions targeting those aspects that are shown here to have the greatest impact on functioning.

One finding of note was that the categories within each component of the ICF were highly inter-connected. Specifically, for *Body functions* a close connection was observed among *b122 Global psychosocial functions, b152 Emotional functions*, and *b160 Thought functions*, after controlling for all other connections. Regarding *Activities and participation*, there was a strong connection between *d240 Handling stress and other psychological demands, d175 Solving problems*, and *d720 Complex interpersonal interactions*, after controlling for all other connections. Concerning the *Environmental factors* component, strong positive connections were found between *e310 Immediate family, e355 Health professionals, e450 Individual attitudes of health professional*s, and *e410 Individual attitudes of immediate family members*, and there was also a strong association among *e570 Social security services, systems and policies* and *e580 Health services, systems and policies*, after controlling for all other associations.

These connections further support the clustering of ICF-CS categories as proposed in the ICF model (7). For example, the close connections between *b122 Global psychosocial functions, b152 Emotional functions*, and *b160 Thought functions*, which all belong to the *Mental functions* chapter of the ICF (7), indicate the relevance of problems representing classical symptoms of schizophrenia, for instance, delusions and hallucinations (e.g., *b160 Thought functions*), negative symptoms such as affective flattening (e.g., *b152 Emotional functions*), and psychological functions (*b122 Global psychosocial functions*) (1). This is also consistent with studies indicating that individuals with schizophrenia show impairment in thought and emotional functions such as emotion perception and expression (35, 36), symptoms that represent common therapeutic targets for health professionals (37, 38).

The community structure analysis identified three distinct clusters in the Brief ICF-CS network, corresponding to the components *Body functions, Activities and participation*, and *Environmental factors*, thus reflecting the theoretical ICF components (7). Furthermore, all but two of the 25 Brief ICF-CS categories (i.e., *b156 Perceptual functions* and *b180 Experience of self and time functions*) were found to belong to their corresponding theoretical ICF component. Taken together, these findings provide further support for the multidimensional structure of ICF-CSs and suggest useful directions for future research into functioning and the validity of the ICF-CSs.

It should be noted, however, that some categories showed weak or no connections with other categories from the same ICF component. For instance, no connections were found between either of the two categories *b156 Perceptual functions* and *b180 Experience of self and time functions* and the other categories that comprise the *Body functions* component, whereas a small connection was found between these two categories and, respectively, the categories *d240 Handling stress* and *d840 Apprenticeship (work preparation)*, which belong to the *Activities and participation* component.

From the network perspective, the absence of an edge between *b156 Perceptual functions* and *b180 Experience of self and time functions* indicates their independence from each other and implies that they are conditionally independent from the other categories of the *Body functions* component to which they theoretically belong, as well as from the other categories in the network, with the exception of the categories *d240 Handling stress* and *d840 Apprenticeship (work preparation)*. From the clinical perspective, these findings suggest that positive symptoms (such as hallucinations or delusions) and those related to an awareness of one's identity may be relevant to the execution of daily life tasks and also influence an individual's participation in certain contexts. Specifically, these symptoms might play an important role in hindering a person's ability to deal adequately with emotions and to enter the labor market.

Centrality measures such as node strength can be used as an indicator of the most important variables in the network (39). A noteworthy finding in the present estimation of centrality was the variability in node strength among the 25 categories of the Brief ICF-CS for schizophrenia. The five most central categories in the Brief ICF-CS network, based on their strength as nodes (see Figure 3, Strength column), comprised two categories from the *Environmental factors* component, namely *e410 Individual attitudes of immediate family members* and *e450 Individual attitudes of health professionals* (ranked 1 and 2), and three categories from *Activities and participation*, namely *d910 Community life, d175 Solving problems*, and *d710 Basic interpersonal interactions* (ranked 3, 4, and 5 out of 25, respectively), each of which showed strong connections with other categories. These findings are in line with the results of previous studies in which these five categories (identified here as being the most central) were among the most frequently reported not only by individuals with schizophrenia but also by experts in the assessment and/or treatment of individuals with this health condition (9–12, 27, 28). This suggests, from the network perspective, that these central categories may be key to understanding the functioning problems experienced by individuals with schizophrenia.

In the clinical context, these five central categories (i.e., *e410 Individual attitudes of immediate family members, e450 Individual attitudes of health professionals, d910 Community life, d175 Solving problems*, and *d710 Basic interpersonal interactions*) would potentially be important for predicting functioning problems in individuals with schizophrenia and may also play a critical role in relation to treatment interventions and outcomes. For instance, the strength centrality of *e450 Individual attitudes of health professionals* confirms the central importance of the relationships between individuals with schizophrenia and mental health professionals, as highlighted by Lauber and colleagues (40), who recommended that professionals should have more personal contact with individuals suffering from mental illness so as to minimize the negative impact of stigmatization on these patients. Similarly, the strength centrality of both *e410 Individual attitudes of immediate family members* and *e450 Individual attitudes of health professionals*, alongside the strong positive connections that were found between these two categories and *e310 Immediate family* and *e355 Health professionals*, corroborates previous research (41–43) emphasizing the need for families and mental health professionals to collaborate in order to facilitate a patient's recovery. In this regard, several studies (44, 45) have suggested that a supportive family environment may be clinically useful for improving functioning and recovery in individuals with schizophrenia. By contrast, greater family burden (e.g., lack of family integration, financial difficulties, and limited opportunities) has been linked to worse functional outcomes, insofar as it may affect the level of support that families are able to provide to an individual with schizophrenia, which in turn impacts that person's functioning (46). As regards the fact that three of the five most central categories (i.e., *d910 Community life, d175 Solving problems*, and *d710 Basic interpersonal interactions*) belong to the *Activities and participation* component, this reflects the findings of Izquierdo et al. (47), who likewise observed that patients with first-episode psychosis experienced more difficulties in participation domains (e.g., joining in community activities).

A further point to note in our analysis is that the category *e410 Individual attitudes of immediate family members* also yielded the highest BEI value, followed in rank order by categories *b160 Thought functions, d760 Family relationships* and *e570 Social security services, systems and policies*. However, the results obtained when applying the case-dropping bootstrap technique indicated that the centrality measures, including node centrality and bridge centrality, should be interpreted with caution because the stability of these indices might be unreliable. This result may be due to the small sample size. Whatever the case, it should be noted that the stability coefficients reported in previous studies that have employed the case-dropping bootstrap technique are usually low (48).

From the network perspective, the current results largely support the integrity of the Brief ICF-CS categories, and they have a number of implications. First, the network analysis adds novel findings to the literature on validation of the Brief ICF-CS for schizophrenia, not least by identifying connections between categories both within and across components of the Brief ICF-CS. Second, the identification of a community structure of strongly connected categories, and especially the three meaningful clusters, provides potentially useful information for clinical practice and intervention. Third, the centrality analysis draws attention to the core problems in functioning experienced by individuals with schizophrenia, in this case from the perspective of professionals from different health fields with experience of treating persons with this disorder. These central problems within the Brief ICF-CS network are therefore the ones that should be especially targeted during assessment and treatment. From a psychometric perspective, the central problems in functioning and the identified clusters could also provide the basis for the development of new instruments for assessing these specific aspects of functioning and which would be sensitive to change when comprehensive programs are applied to improve functioning in individuals with schizophrenia.

This study has certain limitations that warrant consideration. One is that the present findings regarding the central problems in the Brief ICF-CS for schizophrenia are based on the perspective of health professionals from different disciplines, and it is unclear whether the same results would be obtained when considering the perspective of individuals with schizophrenia themselves, or that of their families or caregivers. Future studies should therefore investigate the relevance of different problems in functioning from the perspective of individuals with schizophrenia and their families and caregivers so as to enable comparison with the current network findings. A further potential limitation concerns the small number of health professionals from the African and Eastern Mediterranean WHO regions among participants in the six Delphi studies. There were several reasons for this, including difficulty contacting them due to their limited internet access and the lower number of specialized health professionals in these regions. Nevertheless, the expert panels who took part in the Delphi studies did include representatives from all six WHO regions, and the results obtained largely supported the worldwide validity of the Brief ICF-CS for schizophrenia from an expert perspective.

## Conclusion

In summary, network analysis is a useful approach for exploring the network structure of the Brief ICF-CSs and for identifying the most important problems in functioning within the estimated network. Accordingly, within the Brief ICF-CS network, the specific aspects of functioning with high centrality and relations between ICF categories were identified, highlighting the relevance of these categories in the monitoring and treatment of individuals with schizophrenia. Notably, we found support for a three-component model underlying the Brief ICF-CS for schizophrenia and our results further confirm its validity, insofar as the data on which this analysis is based were acquired by surveying professionals from all six WHO regions. The current study therefore makes a major contribution to the literature on validation of the Brief ICF-CS for schizophrenia by evidencing the underlying network and providing a framework for the design of new comprehensive interventions aimed at improving the functioning of individuals with schizophrenia.

## Data Availability Statement

The raw data supporting the conclusions of this article will be made available by the authors, without undue reservation.

## Ethics Statement

The studies involving human participants were reviewed and approved by the Institutional Review Board Committee of University of Barcelona (IRB00003099). The patients/participants provided their written informed consent to participate in this study.

## Author Contributions

Conceptualization and resources: LN, JG-B, MB, and GG. Methodology: GA and JG-B. Software, formal analysis, and writing—original draft preparation: GA. Validation, investigation, visualization, and writing—review & editing: GA, LN, JG-B, MB, and GG. Data curation: LN and GA. Supervision, project administration, and funding acquisition: JG-B. All authors contributed to the article and approved the submitted version.

## Funding

This research was funded by Spain's Ministry of Economy and Competitiveness, grant number PSI2015-67984, Spain's Ministry of Science, grant number PID2019-109887GB-100, and Agency for the Management of University and Research Grants of the Government of Catalonia, grant number 2017SGR1681.

## Conflict of Interest

The authors declare that the research was conducted in the absence of any commercial or financial relationships that could be construed as a potential conflict of interest.

## Publisher's Note

All claims expressed in this article are solely those of the authors and do not necessarily represent those of their affiliated organizations, or those of the publisher, the editors and the reviewers. Any product that may be evaluated in this article, or claim that may be made by its manufacturer, is not guaranteed or endorsed by the publisher.
