# Supplementary material for: Network Analysis of the Brief ICF Core Set for Schizophrenia
Source: Front Psychiatry. 2022 Jun 17;13:852132. doi: 10.3389/fpsyt.2022.852132 (PMC9247197; doi:10.3389/fpsyt.2022.852132)
Supplement: Supplementary file 1 [file Data_Sheet_1.pdf]

Supplementary Material

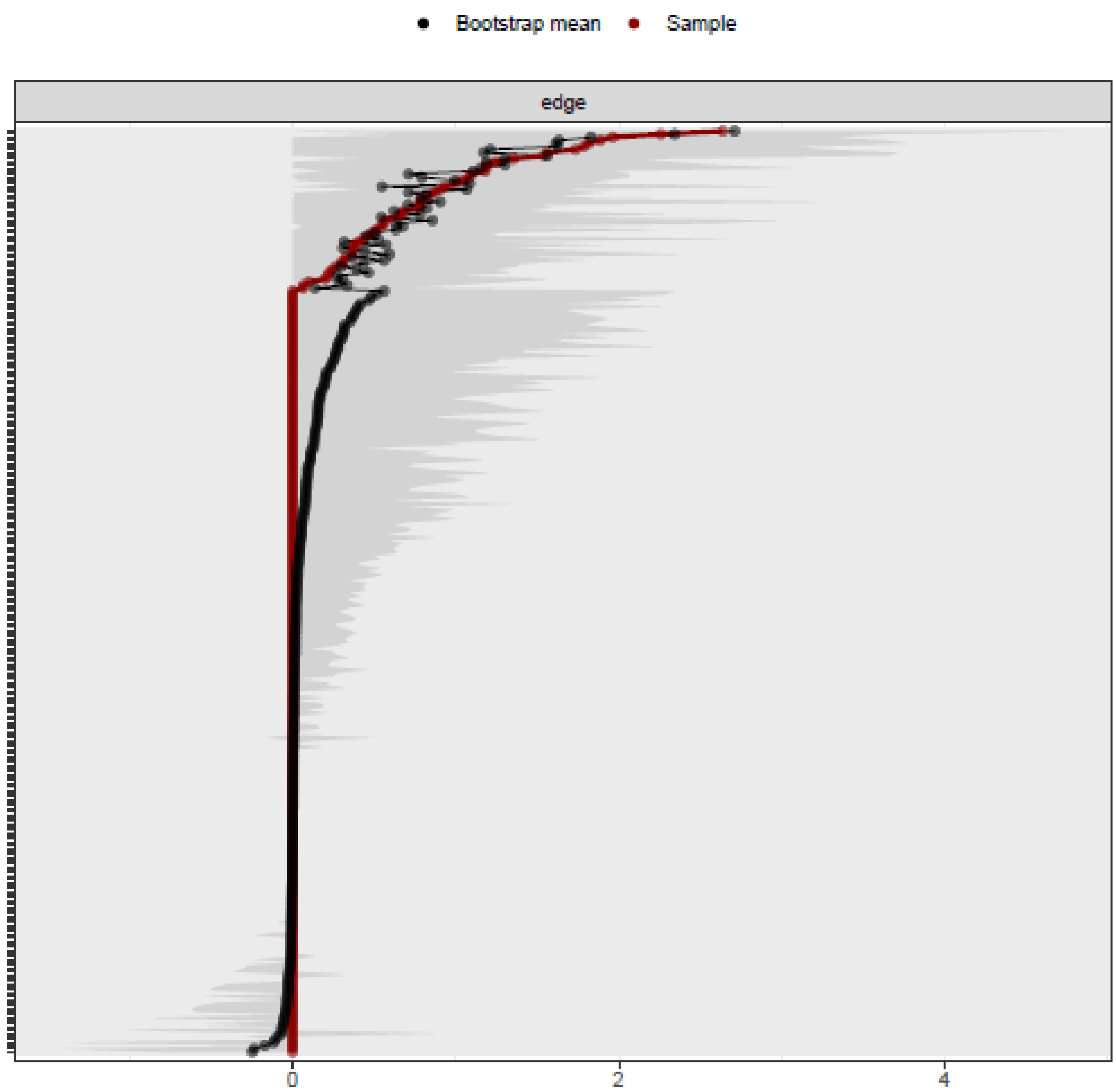

Supplementary Figure S1. Bootstrapped confidence intervals (CIs) of the edge-weights in the 25-category Brief ICF-CS network. The red line represents the edge-weight values, the gray area the 95% CIs, and the black line the bootstrap mean.

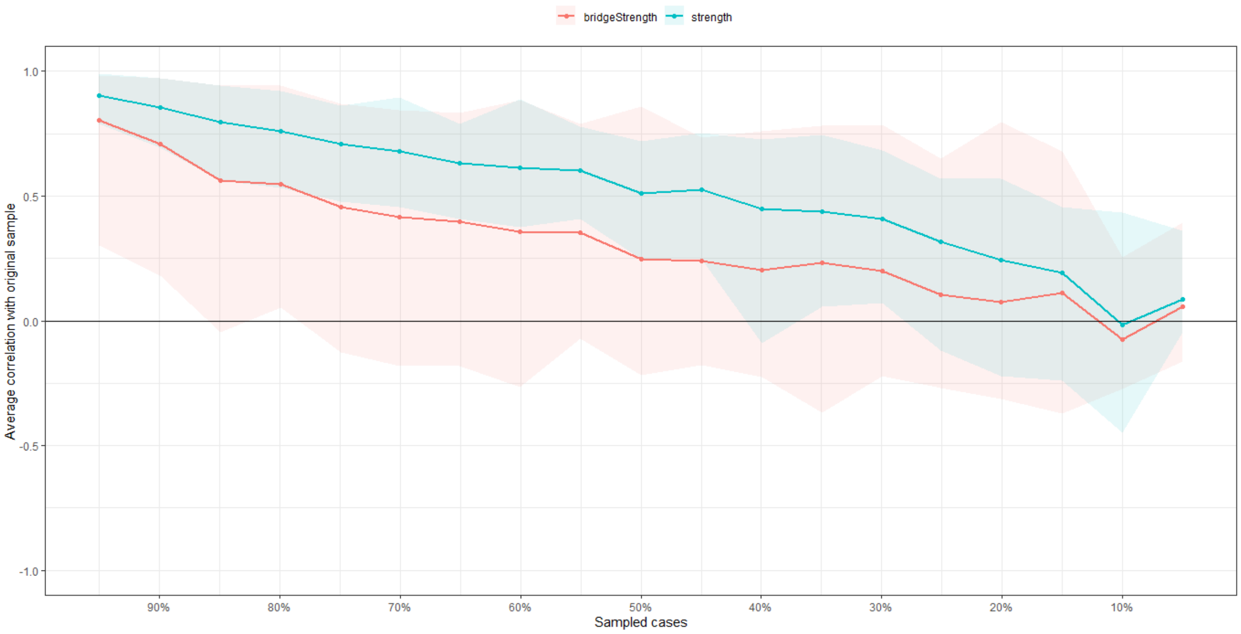

Supplementary Figure S2. Stability of centrality indices (node strength and bridge expected influence) using the case-dropping method.
